# Dark-phase melatonin administration does not reduce blood pressure but induces changes in parameters related to the control of the cardiovascular system in spontaneously hypertensive rats

**DOI:** 10.1038/s41440-025-02247-3

**Published:** 2025-06-09

**Authors:** Hana Mauer Sutovska, Lubos Molcan, Peter Stefanik, Michal Zeman

**Affiliations:** Department of Animal Physiology and Ethology, Faculty of Natural Sciences, Comenius University in Bratislava, Bratislava, Slovakia

**Keywords:** Melatonin, Blood pressure, Telemetry, Spontaneously hypertensive rats, Kidney

## Abstract

Melatonin is synthesised during the dark phase of the day, and its biosynthesis is inhibited by light. Exogenously supplied melatonin has been reported to have hypotensive effects. However, in animal experiments, melatonin is usually administered in one high dose and blood pressure (BP) is almost exclusively measured by plethysmography during the light phase of the day. We tested the effects of melatonin administration in drinking water during the dark phase of the day at different concentrations (2–45 mg/kg/12 h) for three weeks and telemetrically measured haemodynamic variables in spontaneously hypertensive rats (SHR). We measured gene and protein expression in the hypothalamus, brainstem, kidney and adrenal gland. We did not observe a BP decrease even at the highest melatonin concentration. We observed a dose-dependent increase in the percent recovery point and a decrease in dP/dt_max_, particularly during the light phase at lower doses. The effects on the autonomic nervous system and baroreflex were equivocal, with changes observed in both experimental and placebo groups. Melatonin dose-dependently decreased vasopressin expression in the supraoptic nuclei. In the adrenal gland, melatonin increased tyrosine hydroxylase expression. In the kidney, low melatonin doses increased endothelial nitric oxide synthase, while higher doses decreased CD68 levels. Our results do not confirm the hypotensive effects of melatonin in SHR. The potential beneficial effects of melatonin could result from a long-term impact on various organs involved in BP regulation and interaction with multiple molecular pathways, some of which may manifest in improved cardiovascular health in the long term.

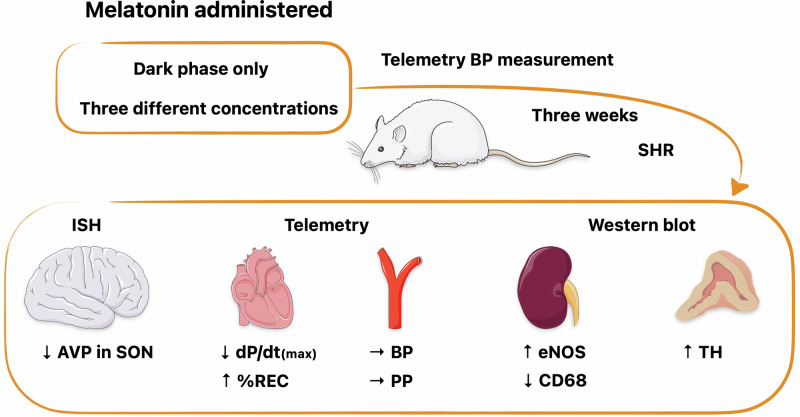

## Introduction

Melatonin is a hormone produced by the pineal gland. It is a pleiotropic molecule that modifies functional and morphological variables of physiological systems, including protective cardiovascular effects in humans and experimental animal models. In diurnal animals, including humans, plasma melatonin levels naturally peak at night, when the blood pressure (BP) is typically low. By contrast, melatonin levels and BP are elevated at night in nocturnal experimental animals such as mice and rats [1]. Cardioprotective effects of melatonin are explained in the literature by its ability to improve the activity of the heart, blood vessels, kidneys and various organs and tissues [2]. The possible mechanisms include its action through receptors in the heart [3, 4] and conduit and resistance blood vessels [5–8]. Additional mechanisms of melatonin action may include: 1) Antioxidant effects, which are frequently highlighted for their role in reducing oxidative injury. Oxidative stress is often associated with endothelial dysfunction and reduced bioavailability of nitric oxide (NO) produced by endothelial NO synthase (eNOS), ultimately impairing NO-mediated vasodilation [9]. Increased oxidative stress is also linked to tissue remodelling, involving key molecules such as transforming growth factor beta 1 (TGF-β1), fibronectin, and collagens. Morphological changes may, in turn, affect renal function and ion homoeostasis, with epithelial sodium channels (ENaC) playing a crucial role. 2) Anti-inflammatory effects, since chronic inflammation of the aorta and kidney contribute to BP dysregulation [10]. The macrophage marker CD68 is commonly associated with vascular and renal inflammation, suggesting a role for immune cell infiltration in these processes [11]. 3) Modulation of the autonomic nervous system [12], which may further influence BP regulation. Melatonin has been implicated in the regulation of tyrosine hydroxylase (TH), a key enzyme in catecholamine biosynthesis, and monoamine oxidase A (MAO-A), an enzyme responsible for the breakdown of neurotransmitters such as norepinephrine and serotonin, thereby influencing sympathetic tone and vascular function [13].

Experimental studies with animals often show hypotensive effects after melatonin administration; however, these studies have limitations. Previous studies often administered melatonin dissolved in water over 24 h, making it practically impossible to determine melatonin intake by one animal during the dark phase, while rats drink water even during the light phase of the day [14]. It is important to consider the dose and test dose-dependent effects. Furthermore, BP is often measured by plethysmography in the light phase of the day [1].

Therefore, we investigated the dose-dependent effects of three week of melatonin administration only during the dark phase of the day on BP and heart rate (HR) in individually housed, freely moving telemetry-measured, adult spontaneously hypertensive rats (SHR). Telemetry allows the evaluation of many variables derived from the pressure curve, which reflects vascular and cardiac functions. Based on beat-to-beat HR and systolic BP variability, we assessed the effect of melatonin on spontaneous baroreflex sensitivity (sBRS) and autonomic nervous system activity. The study takes an exploratory approach, aiming to assess the effects of melatonin on several candidate proteins relevant to BP regulation in the various tissues. In the central regulation, we measured the expression of arginine vasopressin (AVP) in suprachiasmatic nuclei (SCN), supraoptic nuclei (SON) and paraventricular nuclei (PVN), and a component of GABAergic regulation in the nucleus tractus solitarii (NTS). In the periphery, we analysed protein expression of eNOS, ENaC, CD68, TGF- β1 in the kidney and TH and MAO-A in the adrenal glands.

## Materials and methods

### Ethical approval

The Ethical Committee for the Care and Use of Laboratory Animals at Comenius University in Bratislava, Slovak Republic and the State Veterinary Authority of Slovak Republic approved the experiments (Ro-1648/19-221/3). All experiments were conducted per recommendations of the ARRIVE guidelines, Guide for the Care and Use of Laboratory Animals and EU Directive 2010/63/EU for animal experiments.

### Animals

We used SHR (*n* = 34; 317 ± 4 g; RRID:RGD_61000) and normotensive Wistar rats (*n* = 4; 447 ± 16 g; RRID:RGD_597830029) obtained from the breeding station Dobrá Voda, Institute of Experimental Pharmacology and Toxicology, Slovak Academy of Science, Slovak Republic (SK CH 24011). Animals were kept individually in plastic cages under a standard 12-h light (150 lx)/12 h dark (0 lx) regime, at room temperature (21 ± 2 °C) and ambient humidity (55 ± 10%), and given food and water ad libitum.

### Experimental design

At the beginning of the experiment, SHR were held for one week under control conditions without melatonin or placebo treatment. BP was measured continuously telemetrically without manipulating the animals during the measurement. The experiment lasted three weeks, during which bottles were refilled with freshly prepared melatonin daily, according to each animal’s individual water consumption. Melatonin (M5250, Sigma-Aldrich, USA) was diluted in ethyl alcohol (max. 0.5% w/v) in drinking water and available during the dark phase of the day (from 19:00 to 07:00). During the light phase of the day, all animals had access to clean water. We administered melatonin in three doses, and thus divided the SHR into four groups: placebo (tap water with 0.5% ethanol; *n* = 10), Mel2 (melatonin intake: 0.9–2.3 mg/kg/12 h; *n* = 11), Mel10 (melatonin intake 9–10 mg/kg/12 h; *n* = 5) and Mel45 (melatonin intake 41–48 mg/kg/12 h; *n* = 8). Average water intake was approximately five times lower during the light phase than during the dark phase of the day and was stable throughout the experiment (data not shown).

After three weeks, we administered norepinephrine (s.c.; 200 μg/kg; arterenol bitartrate hydrate; Calbiochem, Germany) to the placebo, Mel10 and Mel45 groups two (ZT02; 09:00) and ten (ZT10; 17:00) hours after the beginning of the light phase. At the end of the experiment, the kidneys and adrenal glands were snap frozen in liquid nitrogen, and the brains were immediately removed, placed into cryoprotective medium (Cryomount, Histolab AB, Sweden) and frozen in dry ice. Consequently, tissues were placed at –80 °C for further molecular analyses.

### Blood pressure and heart rate measurement

BP, HR, and locomotor activity were measured by telemetry as described previously [15, 16]. Sensors (TA11 PA-C40; Data Sciences International, MN, USA) were implanted into the flow-restricted abdominal aortas of isoflurane-anaesthetised rats (induction: 4%, maintenance: 1.5–2%; oxygen flow: 1 l/min). After the catheter was inserted, the aorta was sealed with tissue glue (Histoacryl; B. Braun Medical s.r.o., Prague, Czech Republic), and the blood flow was restored. After the surgery, rats were treated subcutaneously with ampicillin (50 mg/kg; BB Pharma a.s., Prague, Czech Republic) and tramadol (15 mg/kg; Tramal, Stada, Bad Vilbel, Germany), kept in a warm place and intensively monitored. Rats without signs of pain, without reduced movement activity and expressing distinct circadian oscillations were included in the experiment approximately 7–10 days after implantation.

### Western blot

The kidney and adrenal glands were homogenised with saccharose buffer containing protease (P8340 Sigma Aldrich) and phosphatase (Sodium orthovanadate and Sodium fluoride) inhibitors. Homogenates were centrifuged twice for 10 min at 3000 rpm and then 11 min at 11000 rpm at 4 °C. The protein concentration was measured using the BCA Protein Assay Kit (Thermo Fisher Scientific, Waltham, MA, USA). Protein lysates (50 ug) were separated by 12% sodium dodecyl-sulphate polyacrylamide gel electrophoresis (Owl P8DS, Owl Separation systems, USA) and transferred to a nitrocellulose membrane. Non-specific bands were blocked with 1% non-fat dry milk or 5% bovine serum albumin (1193004, Serva) in tris-buffered saline with 0.1% Tween® 20 detergent (MP Biomedicals, Eschwege, Germany). Subsequently, nitrocellulose membranes with bound proteins were incubated with the specific primary antibodies: anti-CD68 (1:1000, ab31630, Abcam; RRID:AB_1141557), anti-ENaC (1:1000, ab214192, Abcam; RRID: AB_3676284), anti-eNOS (1:1000, ab76198, Abcam; RRID:AB_1310183), anti-glyceraldehyde-3-phosphate dehydrogenase (1:5000, GAPDH, MAB374, Merck; RRID:AB_2107445), anti-MAO-A (1:1000, ab232845, Abcam; RRID: AB_3676282), anti-TGF-β1 (1:1000, ab215715, Abcam; RRID:AB_2893156) and anti-TH (1:1000, FNab09870, Fine Test; RRID: AB_3676283) and then with the appropriate horseradish peroxidase-conjugated secondary antibodies, anti-mouse (1:1000–1:5000, 7076, Cell Signalling; RRID:AB_330924) or anti-rabbit (1:1000–1:5000, 7074, Cell Signalling; RRID:AB_2099233). The protein signal was detected by chemiluminescence using Clarity Western ECL Substrate (Bio-Rad Laboratories, Hercules, CA, USA) and visualised on the Vü-C chemiluminescence imaging system (Pop-Bio Imaging, Milton, UK; RRID:SCR_026465). Protein expression was quantified using Quantity One Basic software (4.6.6, Bio-Rad Laboratories, Inc., USA; RRID:SCR_014280), and the data were normalised to the expression of GAPDH.

### In situ hybridization

In situ hybridisation was conducted as previously described [17]. Briefly, 14 µm thick coronal sections of brains were sliced at –24 °C using a cryostat (Microm HM520; Germany) and stored at –80 °C. Sections from the anterior hypothalamus and brain stem were warmed to room temperature, fixed with 4% formaldehyde in phosphate-buffered saline (pH 7.4), washed twice in phosphate-buffered saline and subjected to acetylation and delipidation. Oligodeoxyribonucleotide probes, labelled with [35S]dATP, included AVP 48-mer [18], glutamic acid decarboxylase 65 (GAD65) 30-mer [19], glutamic acid decarboxylase 67 (GAD67) 30-mer [20], β2 subunit of gamma-aminobutyric acid A receptor (GABA_A_R) 38-mer [21] and GABA transporter 1 (GAT1) 30-mer [22]. Hybridisation was conducted at 41 °C overnight with specific disintegrations per minute for each probe. Sections were washed multiple times in saline-sodium citrate, twice at room temperature (for 5 min), three times at 55 °C (for 15 min, 15 min and 30 min) and twice more at room temperature (2 × 30 min), and subsequently dehydrated. Sections were then exposed to X-ray film for varying durations, followed by counterstaining with cresyl violet. Optical density signals of AVP mRNA were measured in the SON, PVN and SCN, while GAD65, GAD67, GAT1 and β2 GABA_A_R mRNAs were evaluated in the NTS [23]. Hybridisation signals were analysed using Image J software (v 1.47i NIH, USA; RRID:SCR_003070) and expressed as relative optical densities. The most intense signals from at least six photographs per animal were selected for statistical analysis.

### Data analysis and statistics

Telemetry data were measured continuously (sampling frequency: 500 Hz) for five days (Thursday – Tuesday) for the control and each melatonin administration week. From 5-min segments, outliers higher than three standard deviations were removed, and time- (NN9, number of pairs of successive normal-to-normal intervals that differ by more than 9 ms; pNN9, proportion of NN9 intervals; SDNN, the standard deviation of normal-to-normal intervals; RMSSD, the root mean square of successive differences between normal heartbeats) and frequency-domain features were calculated. Segments for the frequency domain were adjusted (5 Hz interpolation; stationary wavelet transform to decompose the signal, using Daubechies-3 wavelet, into six levels; overlapping 64 of 256 points; resolution 521 points) to calculate low frequency (LF; 0.20–0.75 Hz), high frequency (HF; 0.75–2.5 Hz) and the LF/HF frequency band ratio in HRV Analysis Software (RRID:SCR_026473) [24]. The sBRS was estimated as the α-index within the LF range ((LF_HRV_/LF_BPV_)^0.5) [15, 16].

Additional parameters, such as pulse pressure, the maximum positive (dP/dt_max_) and negative (dP/dt_min_) value of the first derivative of the pressure that occurs during the cardiac cycle, ejection time (time from the rise of the systolic BP to the point dP/dt_min_), time to pressure peak (time from the rise to the peak pressure) and the percent recovery point (the time during which the systolic arterial BP drops to 70% of the pulse pressure value), were extracted from the arterial BP using the proprietary software Ponemah v 6.51 (DSI, Minesota, USA; RRID:SCR_017107).

Using Chronos-Fit software (RRID:SCR_026472), we analysed 24-h oscillations of HR, systolic BP, pulse pressure and locomotor activity expressed as the percent rhythm and amplitude [25].

We calculated the 90-min pressor response to norepinephrine administration as the area under the curve normalised to the basal value (120 min before administration).

We tested the normality of the data using the Shapiro–Wilk test. For a value of *p* < 0.05, we used the Friedman rank sum test; otherwise, Univariate Type III Repeated-Measures ANOVA Assuming Sphericity. We tested Sphericity using Mauchly tests for Sphericity. If Mauchly tests for Sphericity were significant (*p* < 0.05), we used Greenhouse-Geisser and Huynh-Feldt Corrections for Departure from Sphericity. We evaluated and visualised the data using R (version 4.3.2; packages: ggplot2, RRID:SCR_014601; ggpubr, RRID:SCR_021139; ggsci, RRID:SCR_026471; afex, RRID:SCR_022857; emmeans, RRID:SCR_018734; patchwork, RRID:SCR_024826; cowplot, RRID:SCR_018081; ggtext, RRID:SCR_026470) and GraphPad Prism 8.4.3 (GraphPad Software, Inc.; Boston, MA, USA; RRID:SCR_002798). We considered values with *p* < 0.05 to be statistically significant.

## Results

### Heart rate, arterial blood pressure and locomotor activity

**Fig. 1:**
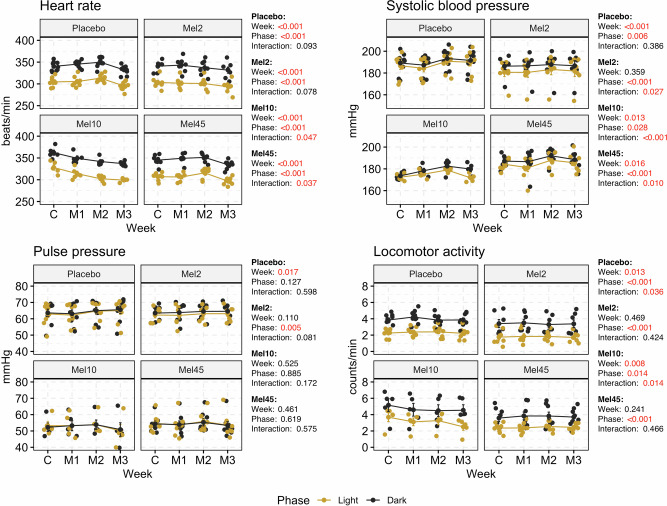
Heart rate, systolic blood pressure, pulse pressure and locomotor activity in rats with nocturnal melatonin administration at three concentrations (Mel2, *n* = 7; Mel10, *n* = 5 and Mel45, *n* = 8) and in the placebo group (*n* = 9). Graphs contain the results of two-way ANOVA. C control week, M1, M2 and M3 first, second and third week with melatonin administration. Data are visualised as individual datapoints and arithmetic mean ± the standard error of the mean

HR and systolic BP, but not pulse pressure, showed a significant difference between the light and dark phases of the day in all groups (Fig. 1). Systolic BP and HR changed significantly between individual weeks in the melatonin-treated and placebo groups, while the placebo and experimental groups did not differ at the end of the experiment during the light (*p* = 0.800; 1-way ANOVA) and dark (*p* = 0.878; 1-way ANOVA) phases of the day. By comparing the control and the last experimental week (M3), we found that the HR was significantly reduced in the light and dark phases of the day in all measured groups (Supplementary Fig. S1 and Supplementary Table S1). For systolic BP, we observed a significant increase between the control and M3 week only in the placebo group in both phases of the day (light: 187 ± 3 mmHg vs 190 ± 3 mmHg, *p* = 0.027; dark: 189 ± 3 mmHg vs 192 ± 3 mmHg, *p* = 0.034; Supplementary Fig. S1 and Supplementary Table S1). In the Mel10 group, we observed a narrowly significant systolic BP increase (*p* = 0.069; 174 ± 2 mmHg vs. 180 ± 2 mmHg) in the dark phase during M3 compared to the control week. Pulse pressure did not change in any of the measured groups.

**Fig. 2:**
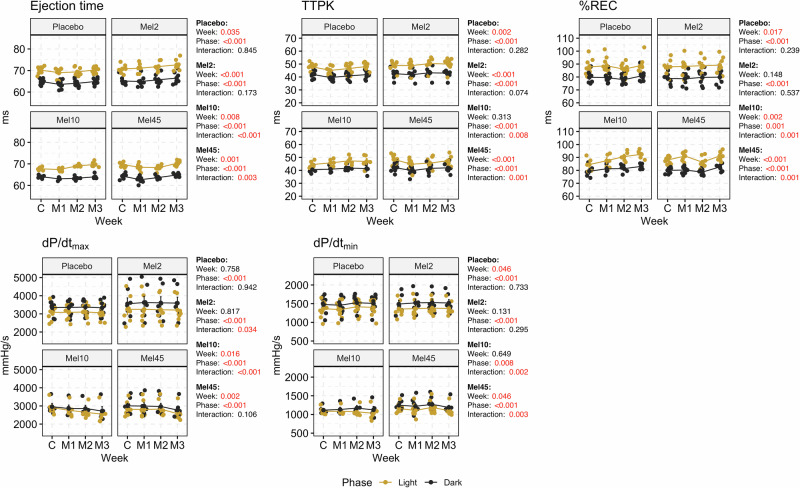
Ejection time, time to pressure peak (TTPK), the percent recovery point (%REC), the maximum positive (dP/dt_max_) and negative (dP/dt_min_) value of the first derivative of the pressure that occurs during the cardiac cycle in rats with nocturnal melatonin administration at three concentrations (Mel2, *n* = 7; Mel10, *n* = 5 and Mel45, *n* = 8) and in the placebo group (*n* = 9). Graphs contain the results of two-way ANOVA. C control week, M1, M2 and M3 first, second and third week with melatonin administration. Data are visualised as individual datapoints and arithmetic mean ± the standard error of the mean

We also observed significant changes in the ejection time, time to pressure peak and the percent recovery point between individual weeks of measurement in almost all groups. We observed significant changes only in dP/dt_max_ and the percent recovery point by comparing the control week and M3 week, for Mel10 only in the light (dP/dt_max_: 2913 ± 187 mmHg/s vs 2542 ± 243 mmHg/s, *p* = 0.006; the percent recovery: 85 ± 2 ms vs 92 ± 2 ms, *p* = 0.017) and for Mel45 in both the light (dP/dt_max_: 2820 ± 135 mmHg/s vs 2573 ± 130 mmHg/s, *p* = 0.003; the percent recovery: 88 ± 1 ms vs 91 ± 1 ms, *p* = 0.031) and the dark (dP/dt_max_: 3003 ± 141 mmHg/s vs 2772 ± 138 mmHs/s, *p* = 0.008; the percent recovery point: 80 ± 1 ms vs 83 ± 1 ms, *p* = 0.015) phases of the day (Fig. 2; Supplementary Table S1).

We observed significant differences in locomotor activity between phases. For the placebo and Mel10 groups, we observed an effect between weeks (Fig. 1), while we only observed a significant decrease in locomotor activity for Mel10 in the light phase comparing the control and M3 week (3.7 ± 0.5 counts/min vs 2.5 ± 0.4 counts/min, *p* = 0.005; Supplementary Table S1).

### Heart rate variability and spontaneous baroreflex sensitivity

**Fig. 3:**
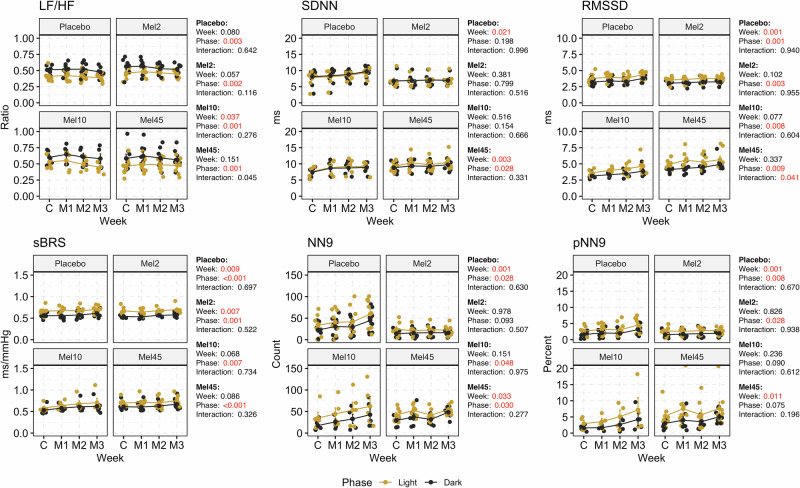
Time- and frequency domain parameters in rats with nocturnal melatonin administration at three concentrations (Mel2, *n* = 7; Mel10, *n* = 5 and Mel45, *n* = 8) and in the placebo group (*n* = 9). Graphs contain the results of two-way ANOVA. C control week, M1, M2 and M3 first, second and third week with melatonin administration, LF low frequency, HF high frequency, sBRS spontaneous baroreflex sensitivity, NN9 number of pairs of successive normal-to-normal intervals that differ by more than 9 ms, pNN9 proportion of NN9 intervals, SDNN the standard deviation of normal-to-normal intervals, RMSSD the root mean square of successive differences between normal heartbeats. Data are visualised as individual datapoints and arithmetic mean ± the standard error of the mean

We observed significant phase differences for variables from the frequency- and time-domain HR variability analysis (Fig. 3). We observed a significant effect of week only in Mel10. By comparing (t-test, Supplementary Table S1) the control and M3 weeks, we showed a significant decrease in the LF/HF ratio in the placebo (0.51 ± 0.02 vs 0.48 ± 0.03, *p* = 0.010) in the dark phase and Mel10 (0.53 ± 0.05 vs 0.45 ± 0.05, *p* = 0.016) in the light phase of the day. Changes in only these two groups and phases were also present in normalised LF (decrease) and normalised HF (increase) (Supplementary Table S2).

In addition, we found significant changes between weeks in the time-domain parameters (SDNN, RMSSD, NN9 and pNN9) in the placebo and Mel45 groups during the measurement. Comparing the control and M3 weeks, we showed a significant increase in SDNN (light: 7.8 ± 0.7 ms vs 9.4 ± 0.4 ms, *p* = 0.045; dark: 8.1 ± 0.8 ms vs 9.7 ± 0.6 ms, *p* = 0.054), RMSSD (light: 3.8 ± 0.3 ms vs 4.4 ± 0.2 ms, *p* = 0.025; dark: 3.2 ± 0.2 ms vs 3.8 ± 0.2 ms; *p* = 0.002), NN9 (light: 33.4 ± 7.6 counts vs 59.3 ± 10.9 counts, *p* = 0.012; dark: 22.5 ± 5.1 counts vs 44.5 ± 8.6 counts, *p* = 0.013) and pNN9 (light: 2.7 ± 0.5% vs 4.6 ± 0.7%, *p* = 0.020; dark: 1.6 ± 0.3% vs 3.1 ± 0.5%, *p* = 0.011) in the placebo group for both phases. Only in the dark phase was this increase observed for NN9 (*p* = 0.004) and pNN9 (*p* = 0.003) in the Mel45 group and for RMSSD (*p* = 0.018) in the Mel10 group. SDNN significantly increased in the Mel10 and Mel45 groups in both phases of the day (Supplementary Table S1).

Significant phase differences in sBRS with higher values during the light phase of the day in all groups and significant variability between individual weeks of measurement were recorded in the placebo (*p* = 0.009) and Mel2 (*p* = 0.007) groups (Fig. 3). Moreover, the t-test showed a substantial increase in sBRS in the dark phase of the day in the placebo (control: 0.56 ± 0.02 ms/mmHg, M3: 0.61 ± 0.03 ms/mmHg, *p* = 0.032) and Mel45 (control: 0.61 ± 0.03 ms/mmHg, M3: 0.67 ± 0.05 ms/mmHg, *p* = 0.039) groups comparing the control and M3 weeks (Fig. 3; Supplementary Table S1; t-tests).

### Noradrenaline administration

The noradrenaline response was higher at the beginning (ZT02; AUC; Placebo group: 434 ± 27 a.u.; Mel10: 384 ± 30 a.u.; Mel45: 395 ± 26 a.u.) than at the end (ZT10; AUC; Placebo group: 322 ± 34 a.u.; Mel10: 316 ± 71 a.u.; Mel45: 309 ± 18 a.u.) of the light phase (repeated 2-way ANOVA *p* = 0.006). Melatonin did not affect the systolic pressor response to norepinephrine (repeated 2-way ANOVA *p* = 0.720).

### Analysis of 24-h rhythm

**Fig. 4:**
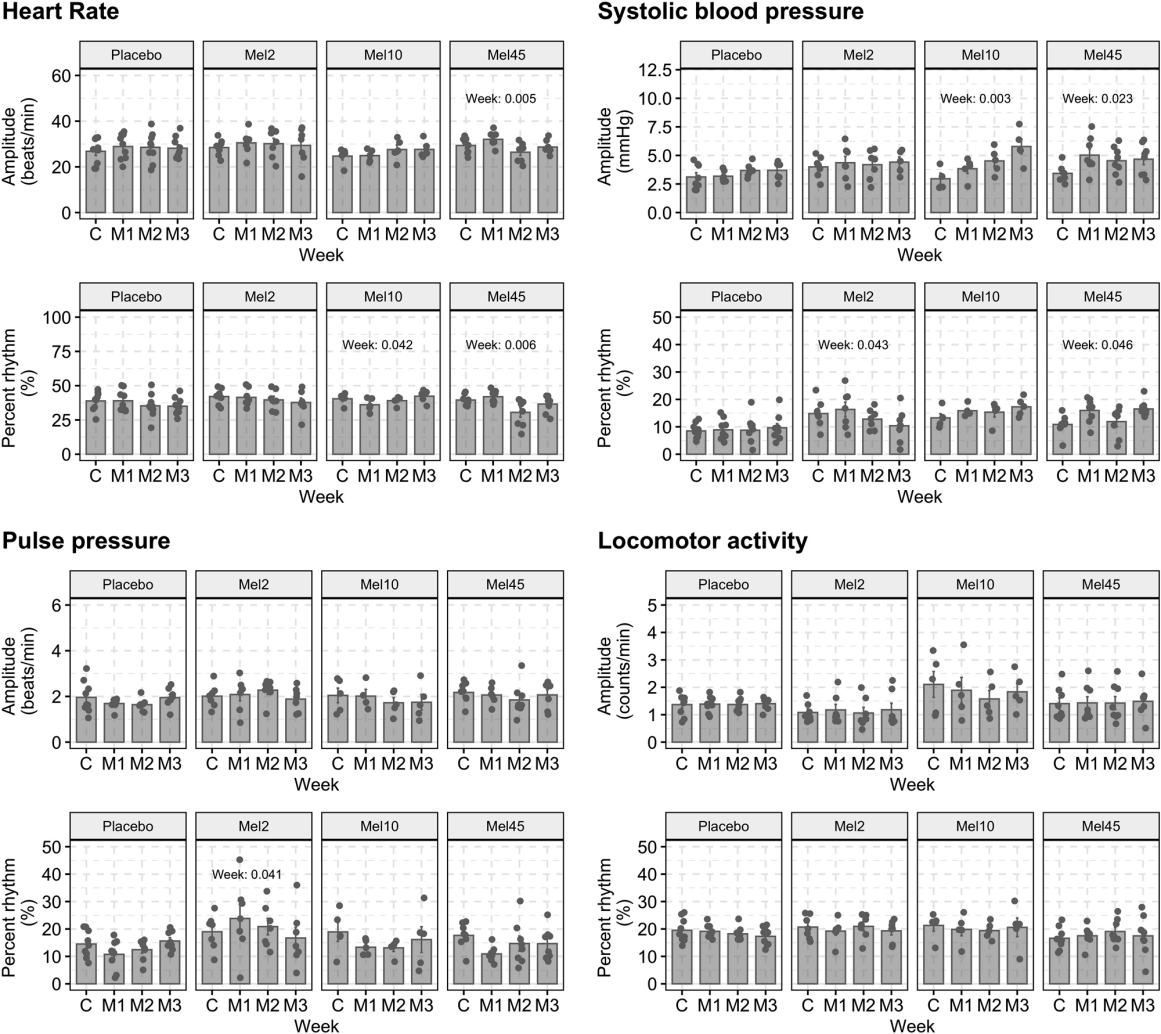
Amplitude and the percent rhythm of heart rate, systolic blood pressure, pulse pressure and locomotor activity in rats with nocturnal melatonin administration at three concentrations (Mel2, *n* = 7; Mel10, *n* = 5 and Mel45, *n* = 8) and in the placebo group (*n* = 9). The graphs contain the results of one-way ANOVA. C control week, M1, M2 and M3 first, second and third week with melatonin administration. Data are visualised as individual datapoints and arithmetic mean ± the standard error of the mean

The amplitude and percent rhythm in the placebo group did not change during the experiment in any of the tested cardiovascular variables (Fig. 4). For systolic BP, we observed a gradual increase in amplitude in the Mel10 (*p* = 0.003) and an immediate increase in the Mel45 (*p* = 0.023) group. We did not observe a change in amplitude in the Mel2 group. The percent rhythm changed in systolic BP in the Mel2 (*p* = 0.043) and Mel45 (*p* = 0.046) groups. We observed an effect of the week in the HR amplitude in the Mel45 (*p* = 0.005) group. The percent rhythm increased in the Mel10 (*p* = 0.042) and decreased in the Mel45 (*p* = 0.006) group. Pulse pressure amplitude and locomotor activity amplitude did not change in any of the measured groups. The percent rhythm in pulse pressure changed only in the Mel2 (*p* = 0.041) group. The percent rhythm in locomotor activity did not change in any of the groups (Fig. 4).

### Molecular analyses

**Fig. 5:**
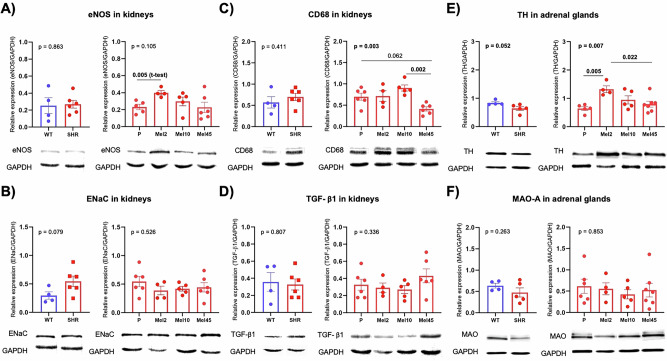
Relative expression of proteins in the kidney (**A**–**D**) and adrenal gland (**E**, **F**) in rats with the nocturnal melatonin administration at three concentrations (Mel2, *n* = 4; Mel10, *n* = 5 and Mel45, *n* = 7) and in the placebo (P, *n* = 6) group. The graphs contain the results of unpaired t-tests (WT, *n* = 4 vs SHR, *n* = 6) and one-way ANOVA. CD68 Cluster of Differentiation 68 – a protein highly expressed by cells in the monocyte lineage (observed 47 kDa), eNOS endothelial nitric oxide synthase (observed 140 kDa), ENaC epithelial sodium channels family (observed 76 kDa), GAPDH glyceraldehyde 3-phosphate dehydrogenase (observed 36 kDa), MAO-A monoamine oxidase A (observed 60 kDa), TGF-β1 transforming growth factor beta 1 (observed 44 kDa), TH tyrosine hydroxylase (observed 60 kDa), WT Wistar rats, SHR spontaneously hypertensive rats. Data are visualised as individual datapoints and arithmetic mean ± the standard error of the mean

In the adrenal gland, SHR had decreased TH (*p* = 0.052), but not MAO-A, compared to Wistar rats. In TH, the Mel2 group had increased expression compared to the placebo and Mel45 groups (Fig. 5). Melatonin did not affect MAO-A. Wistar rats and SHR did not differ in the kidney expression of eNOS, CD68, ENaC and TGF-β1 (Fig. 5A–D). In the Mel45 group, there was a decrease (*p* = 0.062; 1-way ANOVA Tukey post-hoc test) in CD68 compared to the placebo group. The expression of eNOS did not differ between groups, but a comparison of Mel2 with the placebo group showed an increase (*p* = 0.005; t-test) (Fig. 5).

**Fig. 6:**
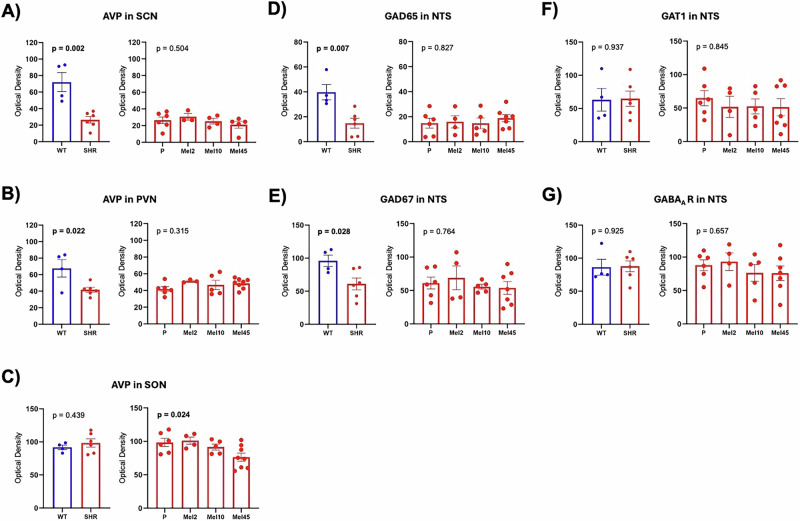
Expression of mRNA in the hypothalamus (**A**–**C**) and brainstem (**D**–**G**) in rats with nocturnal melatonin administration at three concentrations (Mel2, *n* = 4; Mel10, *n* = 5 and Mel45, *n* = 8) and Placebo (P, *n* = 6) group. Graphs contain unpaired t-tests (WT, *n* = 4 vs SHR, *n* = 6) and one-way ANOVA results. AVP arginine vasopressin, GABA_A_R gamma-Aminobutyric acid A receptor, GAD65 glutamic acid, decarboxylase 65, GAD67 glutamic acid decarboxylase 67, GAT1 gamma-aminobutyric acid transporter, NTS the nucleus tractus solitarii, PVN the paraventricular nucleus, SCN the suprachiasmatic nucleus, SON the supraoptic nucleus. Data are visualised as individual datapoints and arithmetic mean ± the standard error of the mean

SHR, when compared to Wistar rats, had significantly decreased AVP expression in the SCN and PVN but not in the SON (Fig. 6A–C). Melatonin did not affect AVP density in the SCN and PVN of SHR but decreased AVP density in the SON in a dose-dependent manner (Fig. 6). Additionally, the density of GAD65 and GAD67 was significantly decreased in SHR compared with Wistar rats (Fig. 6D, E). Melatonin did not affect GAD65 and GAD67 density in SHR in the NTS. GAT1 and GABA_A_R density did not differ between SHR and Wistar rats and was not affected by melatonin treatment (Fig. 6F, G).

## Discussion

### Arterial blood pressure

Several experimental studies have shown significant hypotensive effects of melatonin in rats after only a few weeks when BP was measured by plethysmography and during the light phase of the day (more details in a review [1]). Similar but less clear data have also been published in humans: after melatonin administration, both decreases and increases in BP have been observed [26]. In diurnal animals, including humans, melatonin is generally associated with a nocturnal decline in BP, known as “nocturnal dipping” [27]. This effect can be partially mediated through melatonin’s vasodilatory properties, manifested via activation of MT_1_ and MT_2_ receptors in vascular smooth muscle cells [28], leading to reduced sympathetic tone and increased nitric oxide availability [29]. In contrast, in nocturnal animals, melatonin secretion coincides with their active phase, and the effects on blood pressure can be more complex – vasodilatory or vasoconstrictive responses depending on the receptor distribution in conduit or resistance arteries (endothelial or smooth muscle layer) and physiological state [30]. In the current experiment, we did not observe a drop in telemetrically measured BP, even after three weeks of melatonin administration at a relatively high concentration (45 mg/kg). In stroke-prone SHR measured by telemetry, no changes in BP and HR were observed after two weeks of melatonin administration (s.c.; 0.1 or 1 mg/kg; at the end of the light phase) [31]. Likewise, applying melatonin (1 mg/kg; 30 min before the dark onset) to rats exposed to constant light did not affect telemetrically measured BP [32]. Thus, it seems that the method of BP measurement used is crucial for determining the hypotensive effects of melatonin. Measuring BP by indirect methods causes additive stress, to which the animals become accustomed over time; therefore, plethysmography in the SHR may result in artificially markedly reduced BP [1, 15, 33]. The resulting data can measure habituation rather than the effect of the administered substance.

We observed a significant effect of the week in each group. This suggests that BP exhibited an increasing or decreasing trend or greater week-to-week variability. In hypertensive individuals, increased visit-to-visit BP variability is a risk factor for cardiovascular diseases (reviewed in [34]. Increased short-term BP variability is experimentally presented in increased BP, and the effects vary with the calcium source [35, 36]. Our data showed that melatonin did not affect the week-to-week variability present in many variables and individual groups. Therefore, to identify rising or falling trends in BP in individual groups, we compared the control with the third experimental week. We assumed that the effect of melatonin would be detectable after a longer period of administration despite the week-to-week variability. A comparison of the control and the third experimental week showed a significant increase only in the placebo group, reflecting an increase in BP in almost all rats. In the melatonin-treated groups, we observed interindividual variation, with a decrease, no change or even an increase in BP (Supplementary Fig. S1). In addition to systolic BP, we observed differences between the control and the third experimental week in the HR, ejection time, time to peak and percent recovery point. Differences, albeit smaller, were also observed in locomotor activity, dP/dt_max_ and dP/dt_min_.

### Circadian variability

The daily variability in cardiovascular parameters and locomotor activity is present from the early developmental stages [37] in diverse models [38, 39] and is determined to some extent by the central circadian pacemaker, the SCN [40]. Melatonin synchronises the central oscillator in the SCN [41] and peripheral oscillators, strengthening the daily variability in BP and HR [42, 43]. Our data showed a gradual increase in the amplitude of the systolic BP in the Mel10 group and an immediate rise in the Mel45 group. However, we did not observe these effects on the HR, pulse pressure and locomotor activity. In addition, the 24-h percent rhythm did not increase in any of the evaluated parameters.

Similarly, parameter-dependent results were observed in an earlier experiment, where melatonin administered to rats exposed to constant light restored circadian variability only partially in HR and body temperature, entirely in locomotor activity and not at all in BP. Locomotor activity changes may have resulted from masking to repeated daily subcutaneous melatonin administration 30 min before the start of the dark phase for three weeks [32]. Repeated animal handling and injection cause coupling of animal behaviour and physiological parameters; fear can serve as a non-photic zeitgeber [44].

In the SCN and PVN, we measured the expression of AVP, an important neuropeptide in signal modulation and transmission from the biological clock to the periphery (reviewed in ref. [45]). Some authors explain the mechanism of action of melatonin at the central level by the colocalisation of its receptors with AVP in the SCN, which is the important output signal of the SCN [46, 47]. However, we did not observe the effects of melatonin on AVP in the SCN. Similarly, when melatonin was administered during the dark phase of the day, it did not change the expression of clock genes in the SCN [48]. In addition, we observed a significantly higher expression of APV in normotensive rats than in SHR, in line with the dampened output of the SCN in SHR [49].

Dim light (<5 lx) at night weakens the activity of the central circadian oscillator, reducing the plasma concentration of melatonin [50]. Dim light at night significantly suppresses the circadian amplitude of BP, HR, pulse pressure and dP/dt_max_ [50, 51]. After five weeks of dim light at night, cardiovascular parameters recover depending on the complexity of their regulation [52]. However, the plasma level of melatonin is still reduced [50]. This suggests that if melatonin impacts the 24-h BP amplitude, this effect is not mediated through a central circadian oscillator. However, other regulatory levels of the circadian system may be involved; for example, in the heart, melatonin may alter the functioning of peripheral oscillators, but these effects were not observed in the central oscillator [48]. Melatonin, in contrast to a regular light-dark regime, seems to have only a subtle synchronising effect [40]. Moreover, pinealectomy does not eliminate the circadian variability of locomotor activity [53], suggesting that the effects of melatonin on BP through central circadian effects are questionable.

### Heart rate variability and spontaneous baroreflex sensitivity

To our knowledge, no prior studies have analysed HR variability using continuous telemetry in melatonin-treated SHR. We evaluated autonomic nervous system activity indirectly using time- and frequency-domain analysis of HR beat-to-beat variability. In many cases, the results in the melatonin-treated groups were not consistent, while we also observed changes in the placebo group. Therefore, we cannot state unequivocally that melatonin fundamentally and dose-dependently influenced autonomic nervous activity, which we evaluated indirectly. However, we also confirmed insignificant changes in autonomic nervous activity by applying noradrenaline. By contrast, in the adrenal gland, we observed an increase in tyrosine hydroxylase expression only in the group with a lower dose of melatonin. This could indicate improved synthesis of catecholamines in the adrenal gland. SHR are known to have reduced expression of tyrosine hydroxylase, dopamine beta-hydroxylase and monoamine oxidase A and increased expression of monoamine oxidase B in the adrenal gland compared to normotensive rats [54]. Melatonin was shown to have inconsistent effects on sympathetic activity, varying with dose, tissue, and protocol. Some studies report reduced plasma norepinephrine (not epinephrine) in hypertensive and normotensive rats but improved β1/β2-adrenoceptor ratio only in SHR [55]. Another study found an effect on norepinephrine release from isolated atria only in Wistar (not SHR) rats with high-dose melatonin [56]. Methodological differences, including circadian timing, further complicate comparisons. For example, 24-h electrocardiogram recordings indicated increased HR variability and vagal tone in SHR versus Wistar [57], contrasting with the widely accepted view of sympathetic predominance in SHR [58]. This highlights the importance of context-dependent effects in HR variability interpretation. Notably, suppression of endogenous melatonin by nighttime light exposure [50] was accompanied by decreased LF/HF ratio in both light and dark phases [51], supporting a dynamic, possibly acute link between melatonin and autonomic tone. In our study, melatonin showed inconsistent effects on HR variability but increased adrenal tyrosine hydroxylase at a low dose, suggesting a possible dose-dependent, biphasic effect. Transient BP and HR reductions after low-dose melatonin infusion [59] further support the idea of acute but non-sustained autonomic modulation.

The improvement in baroreflex activity often explains the hypotensive effects of melatonin. Similar to other variables, we observed week-to-week variability in sBRS in the placebo and Mel2 but not in the Mel10 and Mel45 groups. In Mel45, we observed an increase in sBRS sensitivity during the dark phase of the day, which we also observed in the placebo group. In the NTS, we measured the expression of the GAD65, GAD67, GAT1 and GABA_A_R, genes, which are related to inhibitory neuromodulation of the baroreflex. GABA normally acts as a negative modulator of baroreflex activity, tonically reducing sympathetic baroreflex gain in SHR [60]. In the NTS, GABAergic inhibition suppresses excessive baroreflex activity and helps to stabilise BP. Conversely, blocking GABA receptors in the NTS increases baroreflex sensitivity and alters sympathetic output, which may have significant implications for the pathophysiology of hypertension [60]. In SHR, compared to normotensive rats, we showed a decrease in GAD65 and GAD67 but not GABA_A_R, corresponding to increased BP due to sympathetic hyperactivity. Similar data with unchanged GABA_A_R expression, increased GABA_B_ receptor expression and decreased GAD activity in the NTS were observed in Wistar-Kyoto rats with induced hypertension after 5/6 nephrectomy [61]. However, unlike previous studies [59, 62], melatonin administration in our study did not alter baroreflex activity, analysed either as spontaneous sensitivity (sBRS index) or at the molecular level through changes in GAD65 and GAD67 expression in the NTS.

### Kidney associated changes

In kidneys, the expression of eNOS is elevated in young SHR during the development of hypertension [63] but declines with age to levels that are either comparable to [64] or lower than in Wistar rats [65]. Similarly, renal CD68 expression is elevated in young but not in adult SHR [66], consistent with our findings. We demonstrated that melatonin in Mel2 increased the expression of eNOS in the kidney, while in Mel45 it reduced renal macrophage accumulation (CD68). These results suggest that at different concentrations, melatonin could have distinct effects on oxidative damage and inflammation in the kidneys. However, despite these molecular changes, we did not observe a BP decrease in SHR in any of the melatonin groups. Other mechanisms that are often discussed in the treatment of hypertension include the regulation of sodium homoeostasis. Renal ENaC expression rises with age and does not differ between young SHR and Wistar Kyoto rats [67]. Our results indicate an increasing trend (*p* = 0.079) in SHR as compared to Wistar rats, and we observed no changes in the expression of ENaC due to melatonin. On the other hand, in the SON, we did not observe a difference in AVP expression between SHR and Wistar rats, which is in line with previous studies [68, 69]. Other studies have reported either higher [70, 71] or lower [72] AVP levels, depending on the measurement method and the age of the animals. However, in SHR, we measured a dose-dependent decrease in AVP expression in the SON, which may be related to a reduction in circulating vasopressin. In agreement with this, in male Syrian hamsters, an increase in water consumption and urine production without changes in urinary ion concentrations after melatonin administration was observed [73]. AVP in the PVN and SON plays a key role in osmotic homoeostasis; however, no plasma osmolality differences between SHR and Wistar Kyoto rats were reported [74]. Since we also found no differences between SHR and Wistar, hypertension in SHR is likely mediated through alternative pathways. Nevertheless, melatonin’s modulation of SON activity may still be relevant in salt-induced hypertension [75].

We did not observe changes in TGF-β1 in the kidney after three weeks of melatonin treatment. A study with induced fibrosis showed that melatonin reduced collagen accumulation in the aortic media in rats [76] and rabbits [77], possibly decreasing blood vessel stiffness. In our experiment, the effect of melatonin on the arterial dP/dt_max_ was related to the dose. It had no effect in Mel2, changed only during the light phase in Mel10, and changed during both light and dark phases in the Mel45 group, if we compared the control and M3 weeks. We observed similar dose-dependent results for the percent recovery point, i.e. the time until the systolic BP drops to 70% of the pulse pressure value. A change in the length of the percent recovery point can thus indirectly reflect a change in autonomic regulation, pulse BP and vessel stiffness.

### Limitations

We did not measure melatonin in plasma or tissues, nor did we measure its receptors. We only measured water consumption, from which we determined the amount of melatonin the rats received. We also do not know the dynamics of the amount of melatonin the rats received during 12 h of darkness. However, the rats likely drank throughout the dark phase of the day. In humans, controlled-release melatonin is more effective than fast-release melatonin [29]. Therefore, our design, by administering melatonin in water throughout the dark phase, more closely mimicked physiological conditions. The effects of melatonin on the cardiovascular system in diurnal and nocturnal animals may be mediated differently, but underlying mechanisms are not understood, and these differences were not investigated in this study.

SHR exhibit significant variability in measured cardiovascular variables over time. To assess the hypotensive effects of melatonin, it would be advantageous to measure telemetrically BP in other models of hypertension and for a longer time. Exploring these alternative models, including those with borderline hypertension, could provide valuable insights before significant pathophysiological changes are present.

We evaluated the activity of the autonomic nervous system and the sBRS from the indirect analysis of beat-to-beat HR variability. It would be advantageous to have additional data on the pharmacological study of baroreflex sensitivity or direct measurement of nerve activity, such as renal sympathetic nerve activity. However, each of the methods has its limitations. For example, pharmacological administration requires manipulation of the animals, which induces additive stress in the animals. Animals become habituated over time, and the measurements are thus more a result of habituation to the measurement rather than a response to the applied substance. Additionally, excessive manipulation may act as a synchronisation factor, which may bias the measurement of central effects in in vivo models. Measurement of renal sympathetic nerve activity may not reflect overall autonomic nervous system activity. The study was not designed to identify precise mechanisms, and further mechanistic work is needed to determine causal pathways.

## Conclusion

To the best of our knowledge, this is the first study in which melatonin was applied only during the darktime in a dose-dependent manner, and BP was measured by telemetry in freely moving hypertensive rats. After three weeks of melatonin administration, we did not observe hypotensive effects or an alternation in the circadian profile of the BP and HR. Still, at the molecular level, we already showed effects at a low dose of melatonin. We recorded a dose-dependent decrease in AVP in the SON. In the adrenal gland, we observed a reduced expression of TH in SHR compared to Wistar rats, while melatonin increased the expression of TH. In the kidney, we found an increase in eNOS in Mel2, while in Mel45, we recorded a decrease in CD68, indicating potential anti-inflammatory effects. We observed a dose-dependent increase in the percent recovery point and a reduction in dP/dt_max_ at the vascular level, suggesting a mild but significant modulation of cardiovascular regulatory mechanisms by melatonin. Although melatonin did not have a hypotensive effect with dose-dependent administration during the dark phase, it did affect molecular and vascular parameters, highlighting its pleiotropic effects on cardiovascular regulation in hypertensive models.

## Supplementary information

Supplementary material

Supplementary Tables

Supplementary Figure 1

## Data Availability

Data will be made available on request.
