# Supplementary material for: Dark-phase melatonin administration does not reduce blood pressure but induces changes in parameters related to the control of the cardiovascular system in spontaneously hypertensive rats
Source: Hypertens Res. 2025 Jun 9;48(8):2218–33. doi: 10.1038/s41440-025-02247-3 (PMC12321580; doi:10.1038/s41440-025-02247-3)
Supplement: Supplementary file 1 — Supplementary material [file 41440_2025_2247_MOESM1_ESM.docx]

# Supplementary material

**Supplementary Figure S1:** Individual-paired changes in selected variables between control (C) and the third week after the nocturnal melatonin administration (M3) visualised as boxplots. The boxplots display the distribution of individual values for each variable, showing the median (central line), interquartile range (the box representing 50% of the data) and spread of the data (whiskers). Outliers, representing values significantly higher or lower than most of the data, are shown as individual points. These plots provide a clear comparison of the changes in variables such as systolic blood pressure (Sys), heart rate (HR), pulse pressure (PP) and locomotor activity (LA) between the control and melatonin-treated weeks.

**Supplementary Table S1:** Paired t-tests comparing the third week after nocturnal melatonin administration (M3) and the control week were performed for the light and dark phases of the day for blood pressure and blood pressure-derived variables, as well as for the time- and frequency- domain of heart rate variability and spontaneous baroreflex sensitivity.

**Supplementary Table S2:** Comparison of normalised and absolute values of low- (LF) and high- (HF) frequency power bands from spectral and beat-to-beat heart rate variability analysis. C, control week; M1, M2 and M3, first, second and third week of melatonin administration.
