# Supplementary material for: Dark-phase melatonin administration does not reduce blood pressure but induces changes in parameters related to the control of the cardiovascular system in spontaneously hypertensive rats
Source: Hypertens Res. 2025 Jun 9;48(8):2218–33. doi: 10.1038/s41440-025-02247-3 (PMC12321580; doi:10.1038/s41440-025-02247-3)
Supplement: Supplementary file 2 — Supplementary Tables [file 41440_2025_2247_MOESM2_ESM.pdf]

Table S1: paired T-tests (p-values; M3 vs Control week)

Parameter	Group	Light phase	Dark phase
HR	Placebo	0.01453	0.01998
	Mel2	0.00121	0.02882
	Mel10	0.00919	0.00700
	Mel45	0.00213	0.00256
Sys	Placebo	0.02665	0.03387
	Mel2	0.41267	0.78381
	Mel10	0.82697	0.06931
	Mel45	0.49256	0.39338
PP	Placebo	0.01560	0.00532
	Mel2	0.02031	0.03296
	Mel10	0.24378	0.65038
	Mel45	0.56201	0.56282
LA	Placebo	0.76189	0.95355
	Mel2	0.82558	0.80622
	Mel10	0.00513	0.19031
	Mel45	0.34450	0.53350
dP/dt(max)	Placebo	0.52966	0.75099
	Mel2	0.54759	0.46004
	Mel10	0.00608	0.06326
	Mel45	0.00284	0.00802
dP/dt(min)	Placebo	0.05025	0.11987
	Mel2	0.40732	0.07785
	Mel10	0.25881	0.91823
	Mel45	0.08087	0.11704
%REC	Placebo	0.22763	0.14470
	Mel2	0.08142	0.13037
	Mel10	0.01741	0.06217
	Mel45	0.03102	0.01457
ET	Placebo	0.81960	0.93587
	Mel2	0.01413	0.02068
	Mel10	0.03693	0.93568
	Mel45	0.18402	0.21168
TTPK	Placebo	0.65254	0.70838
	Mel2	0.06039	0.22954
	Mel10	0.19069	0.51670
	Mel45	0.07612	0.48256
sBRS	Placebo	0.12532	0.03198
	Mel2	0.24384	0.06197
	Mel10	0.11621	0.06175
	Mel45	0.14102	0.03921

Parameter	Group	Light phase	Dark phase
aLF	Placebo	0.65055	0.05322
	Mel2	0.69662	0.87035
	Mel10	0.92743	0.79579
	Mel45	0.57791	0.67591
aHF	Placebo	0.09621	0.01711
	Mel2	0.99641	0.87237
	Mel10	0.44048	0.26348
	Mel45	0.53934	0.37723
nLF	Placebo	0.55420	0.01348
	Mel2	0.34743	0.22735
	Mel10	0.01262	0.48120
	Mel45	0.61054	0.43318
nHF	Placebo	0.55420	0.01348
	Mel2	0.34743	0.22735
	Mel10	0.01262	0.48120
	Mel45	0.61054	0.43318
LF/HF	Placebo	0.41018	0.00978
	Mel2	0.43404	0.14701
	Mel10	0.01595	0.54838
	Mel45	0.64641	0.39303
NN9	Placebo	0.01238	0.01338
	Mel2	0.81898	0.44272
	Mel10	0.20801	0.05469
	Mel45	0.07140	0.00380
pNN9	Placebo	0.01982	0.01065
	Mel2	0.81180	0.33182
	Mel10	0.27591	0.17240
	Mel45	0.05247	0.00266
RMSSD	Placebo	0.02492	0.00235
	Mel2	0.31866	0.10298
	Mel10	0.12943	0.01831
	Mel45	0.14205	0.06510
SDNN	Placebo	0.04544	0.05419
	Mel2	0.45985	0.25811
	Mel10	0.01020	0.00177
	Mel45	0.02951	0.00769

Table S2: LF and HF, Absolute					
Parameter	Week	Phase	Group	Average	SEM
LF	C	light	Placebo	0.00597	0.00051
			Mel2	0.00591	0.00047
			Mel10	0.00855	0.00111
			Mel45	0.00784	0.00120
		dark	Placebo	0.00565	0.00033
			Mel2	0.00575	0.00046
			Mel10	0.00769	0.00142
			Mel45	0.00857	0.00142
	M1	light	Placebo	0.00638	0.00040
			Mel2	0.00597	0.00049
			Mel10	0.00810	0.00106
			Mel45	0.00919	0.00142
		dark	Placebo	0.00585	0.00044
			Mel2	0.00566	0.00067
			Mel10	0.00760	0.00086
			Mel45	0.00842	0.00143
	M2	light	Placebo	0.00619	0.00046
			Mel2	0.00608	0.00054
			Mel10	0.00803	0.00159
			Mel45	0.00815	0.00085
		dark	Placebo	0.00586	0.00035
			Mel2	0.00582	0.00056
			Mel10	0.00831	0.00188
			Mel45	0.00811	0.00085
	M3	light	Placebo	0.00611	0.00047
			Mel2	0.00597	0.00040
			Mel10	0.00840	0.00241
			Mel45	0.00831	0.00102
		dark	Placebo	0.00628	0.00043
			Mel2	0.00573	0.00047
			Mel10	0.00788	0.00198
			Mel45	0.00885	0.00151

Parameter	Week	Phase	Group	Average	SEM
HF	C	light	Placebo	0.01946	0.00238
			Mel2	0.01665	0.00154
			Mel10	0.01902	0.00258
			Mel45	0.01961	0.00143
		dark	Placebo	0.01312	0.00093
			Mel2	0.01275	0.00091
			Mel10	0.01415	0.00163
			Mel45	0.01670	0.00110
	M1	light	Placebo	0.02076	0.00260
			Mel2	0.01581	0.00108
			Mel10	0.01780	0.00127
			Mel45	0.02113	0.00160
		dark	Placebo	0.01491	0.00213
			Mel2	0.01212	0.00118
			Mel10	0.01307	0.00084
			Mel45	0.01561	0.00121
	M2	light	Placebo	0.02072	0.00300
			Mel2	0.01627	0.00065
			Mel10	0.01939	0.00309
			Mel45	0.02004	0.00166
		dark	Placebo	0.01396	0.00172
			Mel2	0.01328	0.00096
			Mel10	0.01551	0.00231
			Mel45	0.01576	0.00050
	M3	light	Placebo	0.02095	0.00252
			Mel2	0.01665	0.00099
			Mel10	0.02304	0.00506
			Mel45	0.02214	0.00362
		dark	Placebo	0.01670	0.00168
			Mel2	0.01289	0.00070
			Mel10	0.01544	0.00256
			Mel45	0.01996	0.00364

Table S2: LF and HF, Normalised					
**Parameter**	**Week**	**Phase**	**Group**	**Average**	**SEM**
LF	C	light	Placebo	0.26635	0.00833
			Mel2	0.29725	0.01698
			Mel10	0.32343	0.01821
			Mel45	0.29756	0.02024
		dark	Placebo	0.32090	0.00757
			Mel2	0.33961	0.02124
			Mel10	0.35646	0.01537
			Mel45	0.34567	0.02349
	M1	light	Placebo	0.27384	0.01097
			Mel2	0.30471	0.01321
			Mel10	0.33115	0.02749
			Mel45	0.31299	0.01826
		dark	Placebo	0.32214	0.01328
			Mel2	0.33937	0.01905
			Mel10	0.37276	0.02258
			Mel45	0.36078	0.02182
	M2	light	Placebo	0.26939	0.01368
			Mel2	0.29644	0.01204
			Mel10	0.31213	0.01975
			Mel45	0.30838	0.01855
		dark	Placebo	0.32434	0.01498
			Mel2	0.32710	0.01562
			Mel10	0.35641	0.02160
			Mel45	0.35071	0.02061
	M3	light	Placebo	0.26147	0.01014
			Mel2	0.28851	0.01211
			Mel10	0.29002	0.01945
			Mel45	0.30366	0.01953
		dark	Placebo	0.30394	0.01109
			Mel2	0.32431	0.01361
			Mel10	0.34721	0.02596
			Mel45	0.33804	0.02234

Parameter	Week	Phase	Group	Average	SEM
HF	C	light	Placebo	0.73365	0.00833
			Mel2	0.70275	0.01698
			Mel10	0.67657	0.01821
			Mel45	0.70244	0.02024
		dark	Placebo	0.67910	0.00757
			Mel2	0.66039	0.02124
			Mel10	0.64354	0.01537
			Mel45	0.65433	0.02349
	M1	light	Placebo	0.72616	0.01097
			Mel2	0.69529	0.01321
			Mel10	0.66885	0.02749
			Mel45	0.68701	0.01826
		dark	Placebo	0.67786	0.01328
			Mel2	0.66063	0.01905
			Mel10	0.62724	0.02258
			Mel45	0.63922	0.02182
	M2	light	Placebo	0.73061	0.01368
			Mel2	0.70356	0.01204
			Mel10	0.68787	0.01975
			Mel45	0.69162	0.01855
		dark	Placebo	0.67566	0.01498
			Mel2	0.67290	0.01562
			Mel10	0.64359	0.02160
			Mel45	0.64929	0.02061
	M3	light	Placebo	0.73853	0.01014
			Mel2	0.71149	0.01211
			Mel10	0.70998	0.01945
			Mel45	0.69634	0.01953
		dark	Placebo	0.69606	0.01109
			Mel2	0.67569	0.01361
			Mel10	0.65279	0.02596
			Mel45	0.66196	0.02234
