# Supplementary figures and images for: Dark-phase melatonin administration does not reduce blood pressure but induces changes in parameters related to the control of the cardiovascular system in spontaneously hypertensive rats
Source: Hypertens Res. 2025 Jun 9;48(8):2218–33. doi: 10.1038/s41440-025-02247-3 (PMC12321580; doi:10.1038/s41440-025-02247-3)

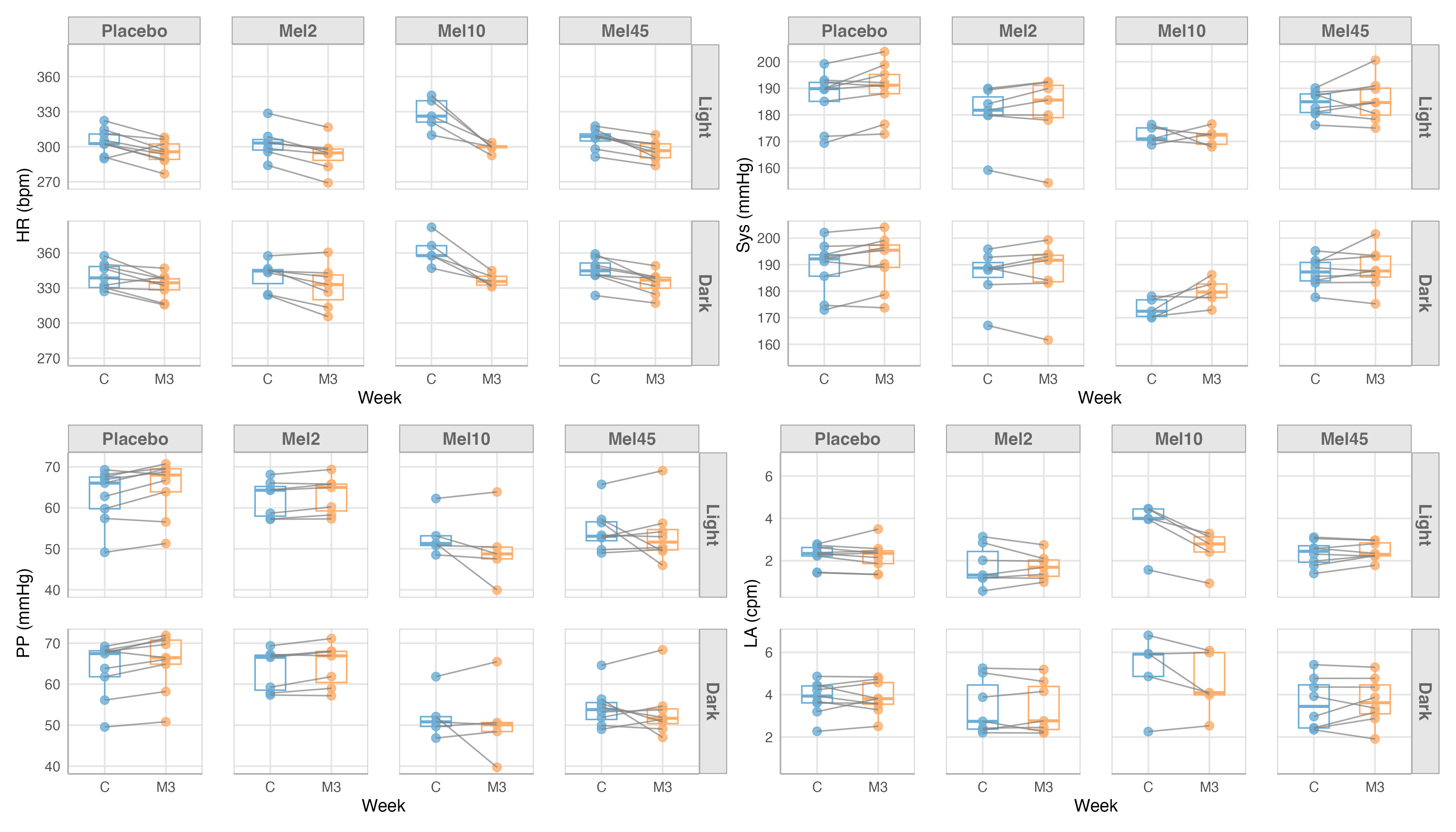

Supplement: Supplementary file 3 — Supplementary Figure 1 [file 41440_2025_2247_MOESM3_ESM.jpg]
